# The Detrimental Effect of Stroke on Motor Adaptation

**DOI:** 10.1177/15459683241309588

**Published:** 2025-01-03

**Authors:** Sabrina J. Abram, Jonathan S. Tsay, Heran Yosef, Darcy S. Reisman, Hyosub E. Kim

**Affiliations:** 1 Department of Psychology, University of California, Berkeley, Berkeley, CA, USA; 2 Helen Wills Neuroscience Institute, University of California, Berkeley, CA, USA; 3 Department of Psychology, Carnegie Mellon University, Pittsburgh, PA, USA; 4 Department of Physical Therapy, University of Delaware, Newark, DE, USA; 5 School of Kinesiology, The University of British Columbia, Vancouver, BC, Canada; 6 Graduate Program in Neuroscience, The University of British Columbia, Vancouver, BC, Canada

**Keywords:** stroke, motor control, motor adaptation, motor impairment, systematic review, meta-analysis

## Abstract

**Background:**

While it is evident that stroke impairs motor control, it remains unclear whether stroke impacts motor adaptation—the ability to flexibly modify movements in response to changes in the body and the environment. The mixed results in the literature may be due to differences in participants’ brain lesions, sensorimotor tasks, or a combination of both.

**Objective:**

We first sought to better understand the overall impact of stroke on motor adaptation and then to delineate the impact of lesion hemisphere and sensorimotor task on adaptation poststroke.

**Methods:**

Following the Preferred Reporting Items for Systematic reviews and Meta-Analyses guidelines, we conducted a systematic review and meta-analysis of 18 studies comparing individuals poststroke to neurotypical controls, with each group consisting of over 200 participants.

**Results:**

We found that stroke impairs motor adaptation (*d* = −0.63; 95% confidence interval [−1.02, −0.24]), and that the extent of this impairment did not differ across sensorimotor tasks but may vary with the lesioned hemisphere. Specifically, we observed greater evidence for impaired adaptation in individuals with left hemisphere lesions compared to those with right hemisphere lesions.

**Conclusions:**

This review not only clarifies the detrimental effect of stroke on motor adaptation but also underscores the need for finer-grained studies to determine precisely how various sensorimotor learning mechanisms are impacted. The current findings may guide future mechanistic and applied research at the intersection of motor learning and neurorehabilitation.

## Introduction

It is indisputable that stroke impairs motor control, as evidenced by symptoms like hemiparesis, spasticity, and loss of independent joint control.^1-10^ Whether stroke impairs motor adaptation, the ability to reduce motor errors through feedback and practice,^11-15^ is controversial. Some studies have found impaired adaptation,^16-18^ while others have found no impairments.^19-21^

Motor adaptation is critical for responding to changes in the body (eg, muscle fatigue) and the environment (eg, walking on uneven terrain). One paradigmatic way to study motor adaptation in the lab is to introduce a perturbation between the motion of the arm and the corresponding visual feedback.^22^ In a typical study, participants are instructed to make reaching movements toward a visual target presented on a horizontally mounted computer monitor.^23^ A visual cursor is also presented on the monitor to indicate hand position, a signal that is readily incorporated into the body schema if its spatial and temporal properties are correlated with the movement. After a few reaches to familiarize the participant with the task environment, a rotation (eg, 45°) is introduced between the motion of the arm and the visual cursor. If participants continued to move directly to the target, the cursor would miss the target, introducing an error. Over several reaches, participants adapt to this perturbation, with the hand’s heading angle shifted in the opposite direction of the rotation (see Methods Section for description of other motor adaptation tasks: force-field adaptation,^24^ split-belt adaptation,^25^ and saccade adaptation^26^). When the perturbation is removed, there are often residual aftereffects in the same direction as learning. Given that motor adaptation refers to changes in feedforward control (ie, how future movements are planned and executed), aftereffects are often regarded as the key signature of learning since it is not influenced by online feedback corrections made during the movement itself.^27^ A change in aftereffect for poststroke individuals compared to controls would indicate that stroke impacts adaptation.

Motor adaptation is critical for maintaining calibrated movement and thus an important focus within neurorehabilitation. Motor adaptation involves various brain areas, including the cerebellum, parietal cortex, motor cortex, and basal ganglia. Damage to any of these areas, such as from a stroke, may, in theory, impair adaptation.^28^ However, it remains unclear whether and to what extent stroke impacts motor adaptation. Once this is determined, it will be important to distinguish between neurorehabilitation strategies aimed at restoring function versus those aimed at compensating for lost function. One systematic review focused on split-belt walking interventions and found that extended training restored step length symmetry in individuals poststroke.^29^ Another study found that improved symmetry transfers to overground walking in individuals poststroke.^30^ The time is ripe to comprehensively evaluate whether and to what extent stroke impairs adaptation, and then apply this knowledge to the design of neurorehabilitation programs.

Impairments in motor adaptation after stroke may depend on the heterogeneity of lesion locations, experimental tasks, or a combination of both. Given that the right and left hemispheres appear to contribute differently to cognition^31^ and motor control,^32^ they may also be differentially involved in adaptation. Indeed, there is evidence pointing toward the selective involvement of the left hemisphere in adaptation.^33^ And given that different experimental tasks may rely on different mechanisms,^34^ the impact of a stroke may also differ between tasks that involve the upper limb (eg, visuomotor adaptation) and those that involve the lower limb (eg, split-belt adaptation).

We performed a meta-analysis to better understand the effect of stroke on motor adaptation. Specifically, we asked whether stroke impacts motor adaptation by synthesizing motor adaptation performance (ie, late adaptation and aftereffect measures) across studies. We also asked whether the impact of stroke on motor adaptation was modulated by the lesioned hemisphere (left vs right) and experimental task (reaching vs walking) by conducting a moderator analysis with these factors as covariates.

## Methods

### Study Selection Criteria

We defined 4 criteria for determining whether studies were included in this meta-analysis: (1) studies included data from poststroke participants as well as age-matched neurotypical controls; (2) outcome measures included those associated with motor adaptation, specifically late adaptation (measured after sufficient experience with a perturbation) and aftereffect (measured immediately after the perturbation was removed); and (3) studies were written in English. At every stage of this systematic review, we adhered to the Preferred Reporting Items for Systematic reviews and Meta-Analyses guidelines.^35^ We use the term “studies” to refer to the sources (papers or posters) identified through our systematic review, and “datasets” to refer to the values input into our meta-analysis. Some studies may contain multiple independent datasets involving different participant groups. For instance, a study might include 2 datasets, one comparing individuals with left hemisphere lesions to a set of controls, and another comparing individuals with right hemisphere lesions to a different set of controls.

### Article Screening

Two authors (JST and HY) independently identified and screened articles from several large databases including the Association for Computing Machinery Digital Library, the Cumulative Index to Nursing and Allied Health Literature, the Cochrane Central Register of Controlled Trials, ProQuest, PubMed, and Scopus. We used the following search terms: implicit OR explicit OR upper extremity OR lower extremity OR paretic OR non-paretic OR dominant OR non-dominant OR subcortical OR cortical OR cortex AND motor learning AND stroke. We did not place strong criteria on the types of movements used to study motor adaptation. However, for studies that involved the lower extremity, we narrowed our inclusion criteria to focus specifically on the most common task: split-belt walking. We also solicited articles from social media, tables of contents from relevant journals (eg, *Neurorehabilitation and Neural Repair*), and citations from related systematic reviews.^29,36^ This review was not pre-registered, and article screening was not blinded. However, SJA and HY independently extracted and entered data from the studies using a double-entry method. We completed our initial search on September 8, 2021, and added new studies until May 14, 2024.

From the eligible datasets, we extracted the following: (1) sample size and average age of patients and controls; (2) lesion hemisphere and lesion location; (3) whether the intact limb or paretic limb was used in the behavioral task; and (4) motor adaptation, that is, the adaptive changes in behavior either during the perturbation block or the aftereffect block. We outline below how we standardized motor adaptation outcomes.

### Data Synthesis and Analysis

We analyzed a range of motor adaptation tasks including those that involved saccades, reaching, and walking. There are 3 common reaching tasks that vary in the nature of the perturbation: in visuomotor rotation tasks, participants reach to a visual target and receive feedback in the form of a visual cursor whose radial distance is matched to the hand but angular distance rotated with respect to the hand.^23^ In visuomotor gain tasks, the visual cursor is perturbed along the radial dimension while the angular distance is matched to that of the hand.^37^ And in force field adaptation tasks, participants reach to a target with a robot arm applying forces to the hand.^24^ In saccade adaptation tasks, participants make eye movements from a start position to a target, where the position of the target jumps immediately upon saccade initiation.^26^ In gait adaptation tasks, the participants walk on a split-belt treadmill with the left and right legs moving at different speeds.^25^

We focused our meta-analysis on 2 possible time points: late adaptation (ie, toward the end of the perturbation block) or aftereffects (ie, immediately after the removal of the perturbation). We standardized measures across datasets with different dependent variables (eg, hand angle, step length asymmetry, and saccade amplitude) by using the mean and standard deviation for patients and controls to calculate Cohen’s *d* and 95% confidence intervals (CI). If the mean and standard deviation were not reported, we calculated the effect size using *F*- or *t*-statistics that compared performance between groups,^38,39^ or estimated the effect size using GRABIT software (Doke, MATLAB Central File Exchange). If a study grouped participants into different categories (eg, mild, moderate, and severe impairment), we estimated the combined mean and standard deviation. For example, in the case of 2 groups, we used the following equations:

(1) $$\mu_{c}=\frac{\left(n_{1}\mu_{1}+n_{2}\mu_{2}\right)}{\left(n_{1}+n_{2}\right)}$$

(2) $$\sigma_{c}=\sqrt{\frac{\left(\begin{array}{l} \left(n_{1}-1\right)\sigma_{1}^{2}+n_{1}\mu_{1}^{2}+\left(n_{2}-1\right)\sigma_{2}^{2}+n_{2}\mu_{2}^{2}\\ -\left(n_{1}+n_{2}\right)\mu_{c}^{2} \end{array}\right)}{\left(n_{1}+n_{2}-1\right)}}$$

Where *n* is the sample size, µ is the mean, σ is the standard deviation, a subscript of *c* represents the combined value, and subscripts of 1 or 2 represent the values for individual groups. We validated this method against simulated data.

We calculated the overall effect size using a random effects model, where the contribution of each dataset was weighted by the sample size and uncertainty in the effect size. We interpreted effect sizes less than 0.2 as small, between 0.2 and 0.8 as medium, and greater than 0.8 as large, and defined the significance level as α = 0.05.^40^ To assess the heterogeneity or variability in effect sizes between datasets, we calculated the *Q* value, which compares the weighted effect sizes to the overall effect size. Smaller *Q* values indicate less variance, while larger *Q* values indicate more variance. To determine whether this variance is greater than that expected by chance, we used Cochran’s *Q* test, which compares the calculated *Q* value to a chi-squared distribution with degrees of freedom equal to the number of datasets minus 1. A significant result (*P* < .05) indicates heterogeneity among effect sizes, meaning the datasets are not consistent with each other. All statistical analyses were performed using R and RStudio, with the data and code available on OSF (https://osf.io/gfmdr/).

### Subgroup Analyses

We conducted subgroup analyses based on 2 covariates: lesion hemisphere and experimental task, restricting these analyses to subgroups with 4 or more datasets. To determine the effect of lesion hemisphere, we compared the datasets that tested individuals with unilateral stroke in the left hemisphere to datasets that tested individuals with unilateral stroke in the right hemisphere. This analysis did not include datasets that combined individuals with left and right hemisphere stroke. For the Wood et al^41^ study, we used available data (https://osf.io/pws2k/) to form left and right hemisphere groups, each matched with a control group in terms of age and sex. Next, to determine the effect of experimental task, we compared visuomotor rotation datasets with split-belt walking datasets. When testing for differences between subgroups, we applied the same approach as when testing for differences between individual datasets. That is, we calculated the *Q* value comparing the subgroup effect sizes to the overall effect size. To determine whether variability between subgroups (left vs right hemisphere lesions or visuomotor rotation vs split-belt walking tasks) is greater than chance, we used Cochran’s *Q* test with degrees of freedom equal to the number of subgroups minus 1.

## Results

**Figure 1.**
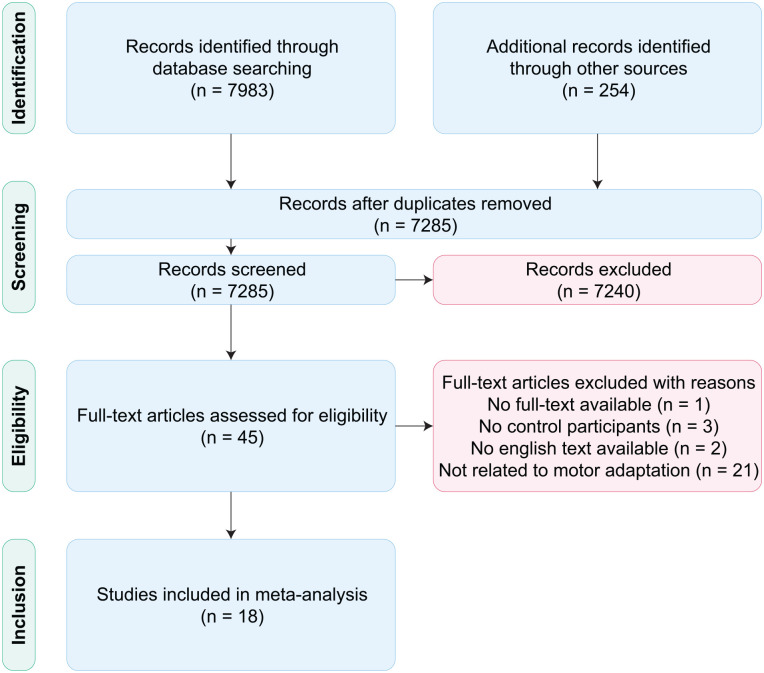
Preferred Reporting Items for Systematic reviews and Meta-Analyses flow diagram describing the study inclusion process of the systematic review. We identified 18 studies, resulting in 21 total datasets, that fulfilled our eligibility criteria.

We included 21 datasets from 18 studies in our meta-analysis (Figure 1). We identified studies using our predefined search terms and then removed duplicates or studies whose title and abstract did not fit our research question. All authors then independently inspected the full text of remaining articles based on our 4 eligibility criteria and agreed upon which articles should be included. We removed 1 study that did not have full text available, 3 that did not include age-matched neurotypical participants, 2 that were not written in English, and 21 that were outside the scope of motor adaptation. Of the 18 remaining studies, 17 were published in peer-reviewed journals or bioRxiv, and 1 was an unpublished dataset.^42^ All data used for this meta-analysis, including the unpublished dataset, as well as detailed study information and methodological information (eg, whether we used GRABIT to extract data from studies), can be found on OSF (https://osf.io/gfmdr/).

**Figure 2.**
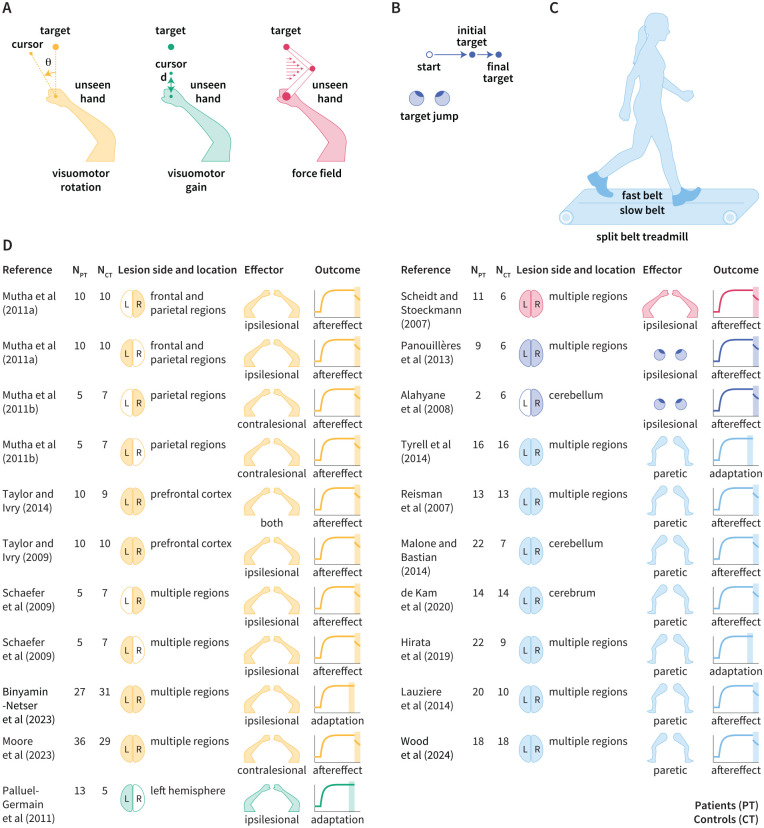
Overview of datasets included in this meta-analysis. Schematic of motor adaptation tasks involving (A) reaching, (B) saccades, and (C) walking. (D) The number of patients (PT) and controls (CT), lesion side (shading indicates left, right, or both; where “both” means that some patients have left hemisphere lesions and others have right hemisphere lesions), and lesion location for each dataset. We also report the effector (eg, arms, legs, eyes), the limb used for the task (contralesional limb, ipsilesional limb, or both; where “both” means that some patients used the contralesional limb and others used the ipsilesional limb), and the outcome measure provided (late adaptation or aftereffect; denoted by the shaded region).^16-21,41-52^

In total, there were 283 participants with stroke and 237 controls. The sample sizes for independent datasets were relatively small (stroke: n = 2-36; control: n = 5-31), motivating a meta-analysis approach to synthesize data across the literature. Of the 21 datasets included in this meta-analysis, 4 measured adaptation only during the perturbation block while 17 measured adaptation during the perturbation block as well as during the aftereffect block (Figure 2). Most of the datasets included individuals in the chronic phase, with an average time since stroke of 4.88 ± 2.72 years across datasets. This value represents a conservative estimate of the time since stroke, as some studies only report the minimum criteria (eg, including patients if the stroke occurred more than 6 months prior). Although we sought to include information on motor impairment levels, such as the Fugl-Meyer Score, this proved difficult due to inconsistent reporting across datasets.

### The Detrimental Effect of Stroke on Motor Adaptation

**Figure 3.**
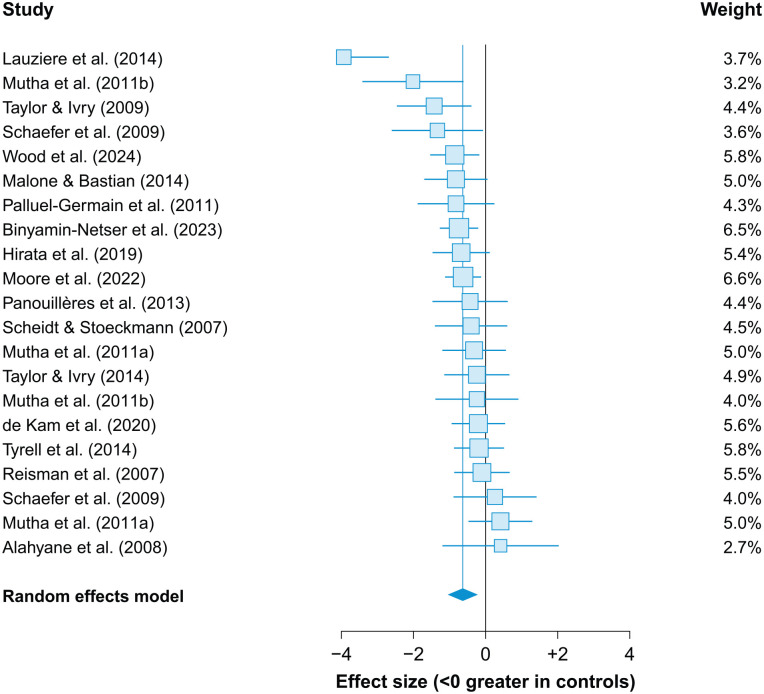
Stroke impairs motor adaptation. Forest plot comparing the performance of individuals poststroke to neurotypical controls, where negative values indicate greater adaptation in controls (ie, impaired adaptation poststroke). The overall effect size is indicated by the blue vertical line. Each square represents a single dataset with its size indicating the weight assigned to that dataset in the random-effects model. Whiskers represent the 95% confidence intervals.

Across 21 datasets, we found that motor adaptation was impaired in individuals poststroke compared to age-matched controls (*d* = −0.63; 95% CI [−1.02, −0.24]; *t*_20_ = −3.40; *P* = .0028; Figure 3 and Supplemental Figure S1 for detailed forest plot). To test the reliability of this finding, we repeated this analysis after excluding 1 outlier (Lauzière et al., 2014) and again found that motor adaptation was impaired in individuals poststroke (*d* = −0.51; 95% CI [−0.73, −0.30]; *t*_19_ = −4.94; *P* < .0001).^49^ We also conducted a risk of bias assessment (Supplemental Figure S3) and found that the results were consistent when the analysis was repeated using only low-risk studies (Supplemental Figure S4). We used Egger’s test to formally assess the presence of a small-study effect and found that the intercept (−0.98 ± 1.19) was not significantly different from zero (*t* = −0.81, *P* = .42), indicating that a small-study effect is unlikely. The funnel plot (Supplemental Figure S5) also shows a symmetrical distribution, supporting this conclusion. We next repeated this analysis for late adaptation, taking into consideration that, in some paradigms (ie, split-belt adaptation), the aftereffect measurement may depend on the initial perturbation, the magnitude of which is not always the same in stroke patients and controls. Late adaptation is not impacted by this issue, as it is compared to baseline. We found that late adaptation was also impaired in individuals poststroke (*d* = −0.43; 95% CI [−0.81, −0.054]; *t*_20_ = −2.38; *P* = .027; Supplemental Figure S2). Lastly, we found that effect sizes varied across these 21 datasets with *I*^2^ = 60.4% (heterogeneity based on χ^2^: *Q*_20_ = 50.56; *P* = .00018), further motivating our subgroup analyses.

### The Impact of Experimental Tasks on Measures of Motor Adaptation Poststroke

**Figure 4.**
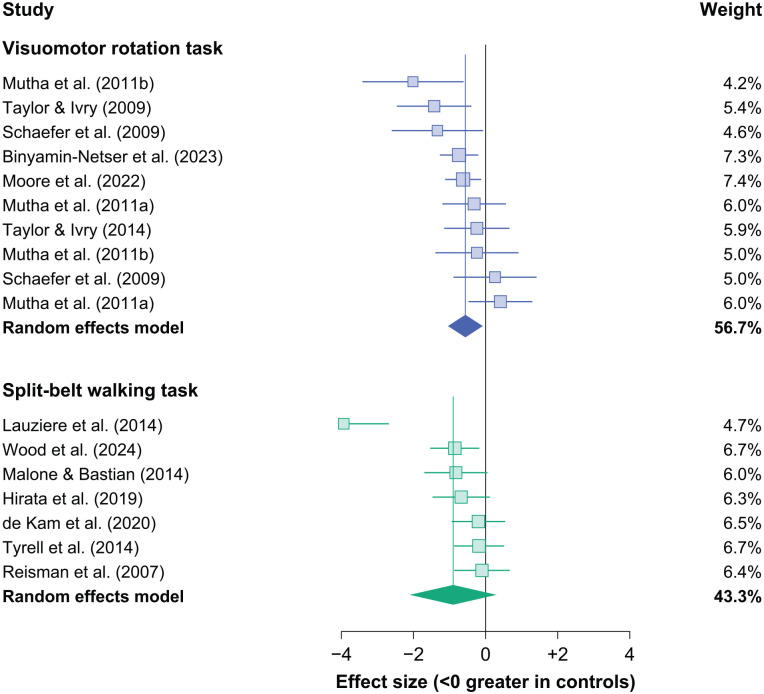
Minimal impact of experimental tasks on motor adaptation poststroke. We assigned subgroups based on whether datasets used visuomotor rotation or split-belt walking tasks. The effect size for each subgroup (purple for visuomotor rotation and green for split-belt walking) is indicated by the vertical line. Each square represents a single dataset with its size indicating its weight and whiskers representing 95% confidence intervals.

We analyzed ten visuomotor rotation datasets (n = 123 patients) and 7 split-belt walking datasets (n = 125 patients) and did not find any significant differences in motor adaptation poststroke between these 2 tasks (*Q*_1_ = 0.41; *P* = .52), suggesting that differences between tasks are not the underlying driver of mixed results in the literature. When examining each task in isolation, the results were less robust as compared to our analysis of all tasks combined. However, we still observed medium-to-large effect sizes related to the negative impact of stroke on adaptation in both visuomotor rotation (*d* = −0.56; 95% CI [−1.03, −0.089]; *t*_9_ = −2.69; *P* = .025; Figure 4) and split-belt walking (*d* = −0.90; 95% CI [−2.08, +0.28]; *t*_6_ = −1.87; *P* = .11; Figure 4) tasks.

### The Impact of Lesion Hemisphere on Measures of Motor Adaptation Poststroke

**Figure 5.**
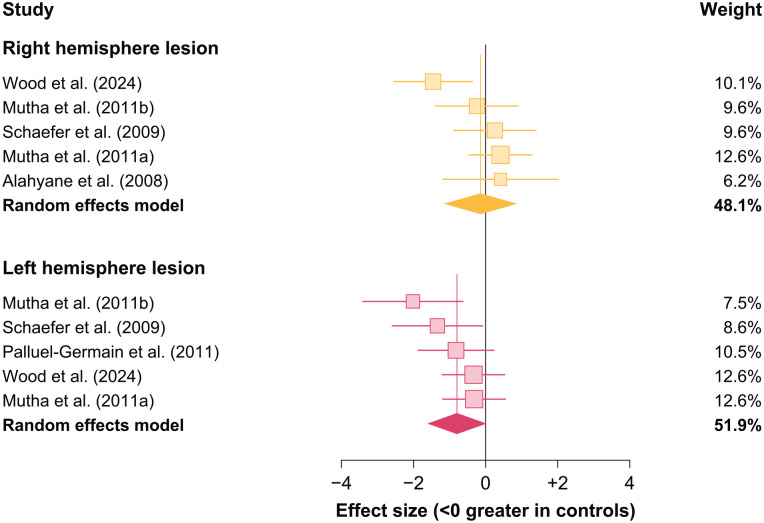
Impact of lesion hemisphere on motor adaptation poststroke. We assigned subgroups based on whether datasets compared motor adaptation between individuals with left hemisphere lesions or right hemisphere lesions against controls. The effect size for each subgroup (red for left hemisphere and orange for right hemisphere) is indicated by the vertical line. Each square represents a single dataset with its size indicating its weight and whiskers representing 95% confidence intervals.

While nearly all studies in this meta-analysis tested individuals with unilateral stroke, many did not examine the impact of lesion hemisphere on motor adaptation (ie, left vs right hemisphere lesions). Here, we analyzed 5 datasets that tested individuals with unilateral stroke in the left hemisphere (n = 43 patients) and 5 datasets that tested individuals with unilateral stroke in the right hemisphere (n = 30 patients). We observed no impairment in individuals with right hemisphere lesions (*d* = −0.14; 95% CI [−1.14, +0.86], *t*_4_ = −0.39, *P* = .71; Figure 5), while there was a substantial (based on Cohen’s *d*), but statistically non-significant, impairment in individuals with left hemisphere lesions (*d* = −0.79; 95% CI [−1.61, +0.027], *t*_4_ = −2.69, *P* = .055; Figure 5). When we performed a direct comparison of motor adaptation with left versus right hemisphere lesions, we also observed a relatively large effect of hemisphere (*d* = −1.11; 95% CI [−2.68, 0.45]), albeit one that was not statistically significant (*Q*_1_ = 1.97, *P* = .16).

## Discussion

We conducted a comprehensive review to examine the impact of stroke on motor adaptation. By synthesizing 21 datasets involving over 200 individuals poststroke, we found that motor adaptation was impaired in individuals poststroke compared to neurotypical controls. Notably, impairments in motor adaptation were consistent across various sensorimotor tasks. When investigating the effect of lesion hemisphere, we found that adaptation remained intact in individuals with right hemisphere lesions, while there appeared to be a sizable (based on Cohen’s *d*), though statistically non-significant, impairment in individuals with left hemisphere lesions. These results motivate further investigation into the impact of lesion hemisphere on motor adaptation.

### Investigating the Impact of Stroke on Implicit Recalibration and Explicit Strategies

Our meta-analysis not only clarified the detrimental impact of stroke on motor adaptation but also identified an important gap in our understanding: *how* does stroke impact the different learning processes supporting motor adaptation? Here, we focus on upper extremity tasks, such as visuomotor adaptation, as it is well established that multiple learning processes support changes in this behavior.^53-56^ There is more work to be done to determine whether these same processes contribute to adaptation in lower extremity tasks such as split-belt walking. For upper extremity tasks, two of the most salient processes include *implicit recalibration*, which keeps our motor system well-calibrated in a gradual and subconscious manner, and *explicit strategies*, which, in contrast, enables rapid and volitional motor corrections.^22^ Existing studies have not cleanly examined the impact of stroke on each process.

**Figure 6.**
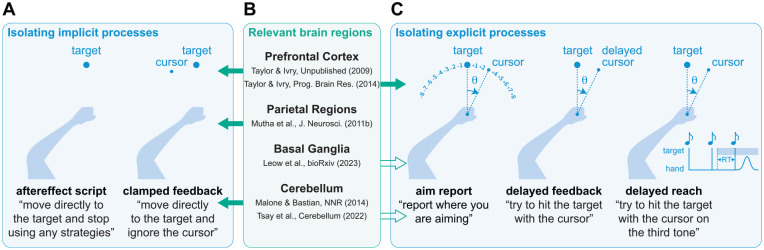
(A) Methods for isolating implicit recalibration. (B) Evidence suggesting which brain regions are involved in which learning processes.^16,18,42,43,64,65^ The solid arrows indicate studies that involve populations with stroke and open arrows indicate studies that involve populations with progressive neurodegenerative disorders. (C) Methods for isolating explicit re-aiming strategies.

There are many experimental methods for dissociating the processes that underlie adaptation, which will be especially useful for identifying how stroke impacts these processes.^22,57-62^ The first method for isolating implicit recalibration involves clearly instructing participants immediately prior to aftereffect trials, in which the perturbation is removed, to forgo any explicit strategies they may have been using (Figure 6A). While most of the studies included in this meta-analysis measured aftereffects, we could not ascertain whether proper instructions were provided (eg, “Move your hand directly to the target and do not aim away from the target.”). Interestingly, a recent study that provided proper instructions found no effect of stroke on implicit recalibration.^21^ Given the singular nature of this study, it will be enlightening to re-examine how stroke impacts this implicit process after several more studies adopt this approach. The second approach is to use clamped visual feedback.^22,59^ Unlike standard visuomotor rotation tasks, the clamped visual cursor moves at a fixed angle away from the target and is not contingent on the participant’s hand angle (Figure 6A). Critically, we inform participants of this manipulation and instruct them to always reach directly to the target and ignore the clamped visual feedback. Despite these instructions, participants exhibit robust implicit recalibration, with learning occurring outside of conscious awareness.^63^

Paralleling the methodological advances for studying implicit recalibration, new approaches have been developed to examine explicit re-aiming strategies. One approach involves asking participants to indicate where they are aiming prior to each reach, revealing the dynamics of explicit re-aiming (Figure 6C).^43,63^ Another approach involves delayed rotated feedback, a manipulation that robustly attenuates implicit recalibration and thus isolates explicit re-aiming (Figure 6C).^66^ A final approach involves manipulations of preparation time. When manipulating the amount of time a visual target is presented before a reach begins, longer preparation time enables the use of explicit strategies that are resource-demanding, whereas shorter preparation time limits deliberation and minimizes the contribution of these explicit strategies (Figure 6C).^67-69^ Taken together, upper extremity tasks provide well-controlled methods for isolating implicit and explicit processes, which are either not present or not easily isolated in other experimental paradigms. Future studies using fine-grained experimental and computational methods to evaluate these processes in individuals poststroke will shed light on the neural correlates of different sensorimotor learning mechanisms (Figure 6B).

### The Role of the Left Hemisphere in Motor Adaptation

The left hemisphere may play a critical role in motor adaptation. Inspired by the literature on brain lateralization, we propose 2 possible mechanisms. First, the left hemisphere may contribute to motor adaptation through feedforward mechanisms.^32,33^ Consequently, participants with left hemispheric stroke may struggle to use prediction errors to update an already faulty motor plan. Second, the left hemisphere may aid motor adaptation through explicit reasoning,^22,70^ particularly in evaluating different action-outcome relationships (eg, rotation, gain, translation).^71,72^ Consequently, participants with left hemispheric stroke may have difficulty pinpointing the true nature of the perturbation, and therefore implementing a control policy to counteract it. Future studies can test these ideas by examining how left hemispheric stroke affects implicit and explicit motor adaptation mechanisms, using tasks that isolate these 2 processes.

However, it is important to note that, although there was a large standardized effect size related to left hemisphere-specific impairment, this finding was statistically non-significant. We offer a few caveats to this finding. First, the lack of robustness may be due to low statistical power, with only 5 datasets in each group (Figure 5). Only a few studies specifically divided stroke participants into different lesion hemisphere groups, and moreover, lesion locations are often not detailed in the manuscript. Therefore, future research using methods such as lesion-symptom mapping is needed to examine how the lesion side (eg, left vs right) and lesion location (eg, subcortical vs cortical) contribute to motor adaptation.

Second, the limb used for the reaching task was often not controlled for, complicating the interpretation of the findings. Ideally, inferences about hemispheric lateralization should be based on studies where the contralesional limb is used for the reaching task. However, out of the ten datasets examining hemispheric contributions, only one had participants use their contralesional limb, with most studies preferring the ipsilesional limb. This preference is understandable given the severe motor control impairments in the contralesional limb, which could make completing the task prohibitive and potentially introduce additional confounds such as fatigue. While it is straightforward to classify which limb was used for reaching tasks, it is not possible to make a definitive classification for other tasks, such as split-belt walking, where both limbs are involved. Since split-belt walking involves one leg on a fast belt and the other on a slow belt, these studies often specify which belt the contralesional limb is placed on. Future studies are needed to examine hemispheric specificity in a more rigorous manner by considering both lesion hemisphere (left vs right) and the limb used (contralesional vs ipsilesional).^73^

### From Fundamental Learning Mechanisms to Targeted Rehabilitation Strategies

A better understanding of how stroke impacts motor adaptation may lead to targeted rehabilitation interventions that are tailored to the specific motor deficits and affected brain regions. With impaired feedforward motor adaptation, rehabilitation therapists could leverage unimpaired learning mechanisms such as learning via explicit instruction^74-76^ and/or reinforcement feedback.^2,14,77,61^ Specifically, therapists might engage the patient in a motor task relevant to their daily life, offering several possible explicit solutions to achieve an important goal, and reward the patient once the goal is achieved. Of course, these compensatory strategies must also be balanced with task-specific training that seeks to restore lost function, in this case, motor adaptation.^2,74,75^ However, if feedforward processes remain intact for some individuals poststroke, therapists could consider interventions that capitalize on this ability.^20,30,76^ That is, if a patient struggles with balanced walking, therapists might introduce a sensorimotor perturbation, such as a weighted vest or uneven terrain, to help the patient implicitly adapt to their motor errors through practice.

Regardless of the exact therapeutic approach, we anticipate that finer-grained neuropsychological research examining how different lesion locations impact various motor learning mechanisms will critically inform future rehabilitation strategies aimed at repairing or remodeling affected neural circuits.^13,78,79^

## Supplemental Material

sj-docx-1-nnr-10.1177_15459683241309588 – Supplemental material for The Detrimental Effect of Stroke on Motor Adaptation Supplemental material, sj-docx-1-nnr-10.1177_15459683241309588 for The Detrimental Effect of Stroke on Motor Adaptation by Sabrina J. Abram, Jonathan S. Tsay, Heran Yosef, Darcy S. Reisman and Hyosub E. Kim in Neurorehabilitation and Neural Repair

sj-eps-2-nnr-10.1177_15459683241309588 – Supplemental material for The Detrimental Effect of Stroke on Motor Adaptation Supplemental material, sj-eps-2-nnr-10.1177_15459683241309588 for The Detrimental Effect of Stroke on Motor Adaptation by Sabrina J. Abram, Jonathan S. Tsay, Heran Yosef, Darcy S. Reisman and Hyosub E. Kim in Neurorehabilitation and Neural Repair

sj-eps-3-nnr-10.1177_15459683241309588 – Supplemental material for The Detrimental Effect of Stroke on Motor Adaptation Supplemental material, sj-eps-3-nnr-10.1177_15459683241309588 for The Detrimental Effect of Stroke on Motor Adaptation by Sabrina J. Abram, Jonathan S. Tsay, Heran Yosef, Darcy S. Reisman and Hyosub E. Kim in Neurorehabilitation and Neural Repair

sj-eps-4-nnr-10.1177_15459683241309588 – Supplemental material for The Detrimental Effect of Stroke on Motor Adaptation Supplemental material, sj-eps-4-nnr-10.1177_15459683241309588 for The Detrimental Effect of Stroke on Motor Adaptation by Sabrina J. Abram, Jonathan S. Tsay, Heran Yosef, Darcy S. Reisman and Hyosub E. Kim in Neurorehabilitation and Neural Repair

sj-eps-5-nnr-10.1177_15459683241309588 – Supplemental material for The Detrimental Effect of Stroke on Motor Adaptation Supplemental material, sj-eps-5-nnr-10.1177_15459683241309588 for The Detrimental Effect of Stroke on Motor Adaptation by Sabrina J. Abram, Jonathan S. Tsay, Heran Yosef, Darcy S. Reisman and Hyosub E. Kim in Neurorehabilitation and Neural Repair

sj-eps-6-nnr-10.1177_15459683241309588 – Supplemental material for The Detrimental Effect of Stroke on Motor Adaptation Supplemental material, sj-eps-6-nnr-10.1177_15459683241309588 for The Detrimental Effect of Stroke on Motor Adaptation by Sabrina J. Abram, Jonathan S. Tsay, Heran Yosef, Darcy S. Reisman and Hyosub E. Kim in Neurorehabilitation and Neural Repair

sj-eps-7-nnr-10.1177_15459683241309588 – Supplemental material for The Detrimental Effect of Stroke on Motor Adaptation Supplemental material, sj-eps-7-nnr-10.1177_15459683241309588 for The Detrimental Effect of Stroke on Motor Adaptation by Sabrina J. Abram, Jonathan S. Tsay, Heran Yosef, Darcy S. Reisman and Hyosub E. Kim in Neurorehabilitation and Neural Repair

sj-eps-8-nnr-10.1177_15459683241309588 – Supplemental material for The Detrimental Effect of Stroke on Motor Adaptation Supplemental material, sj-eps-8-nnr-10.1177_15459683241309588 for The Detrimental Effect of Stroke on Motor Adaptation by Sabrina J. Abram, Jonathan S. Tsay, Heran Yosef, Darcy S. Reisman and Hyosub E. Kim in Neurorehabilitation and Neural Repair
