# Supplementary material for: The Detrimental Effect of Stroke on Motor Adaptation
Source: Neurorehabil Neural Repair. 2025 Jan 3;39(3):213–25. doi: 10.1177/15459683241309588 (PMC11921215; doi:10.1177/15459683241309588)
Supplement: sj-docx-1-nnr-10.1177_15459683241309588 – Supplemental material for The Detrimental Effect of Stroke on Motor Adaptation [file sj-docx-1-nnr-10.1177_15459683241309588.docx]

**Supplemental Material**


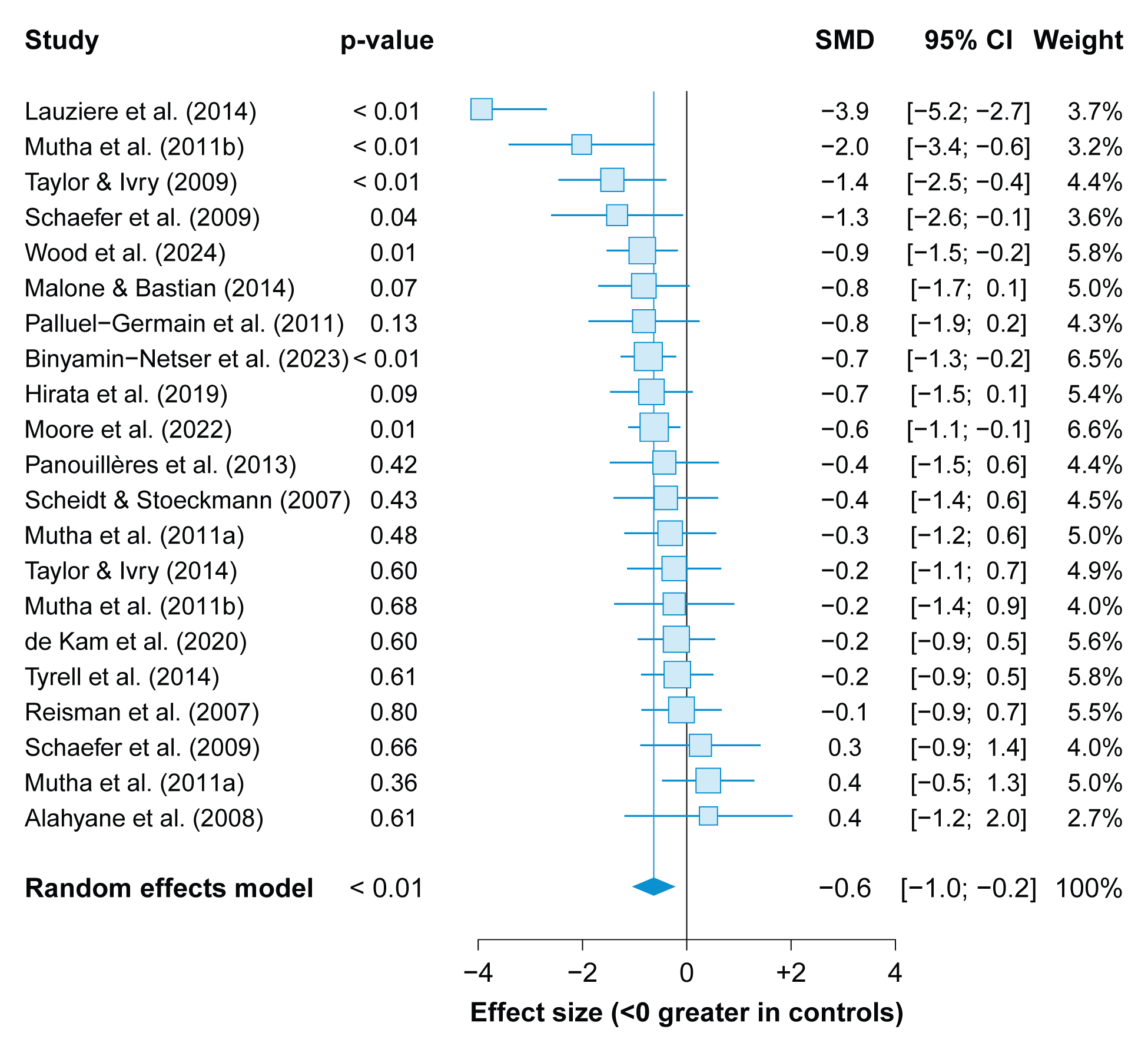


**Figure S1.** Detailed forest plot comparing the performance of individuals poststroke to neurotypical controls, where negative values indicate greater adaptation in controls (i.e., impaired adaptation poststroke). The overall effect size is indicated by the blue vertical line. Each circle represents a single dataset with its size indicating the weight assigned to that dataset in the random-effects model. Whiskers represent the 95% confidence intervals.


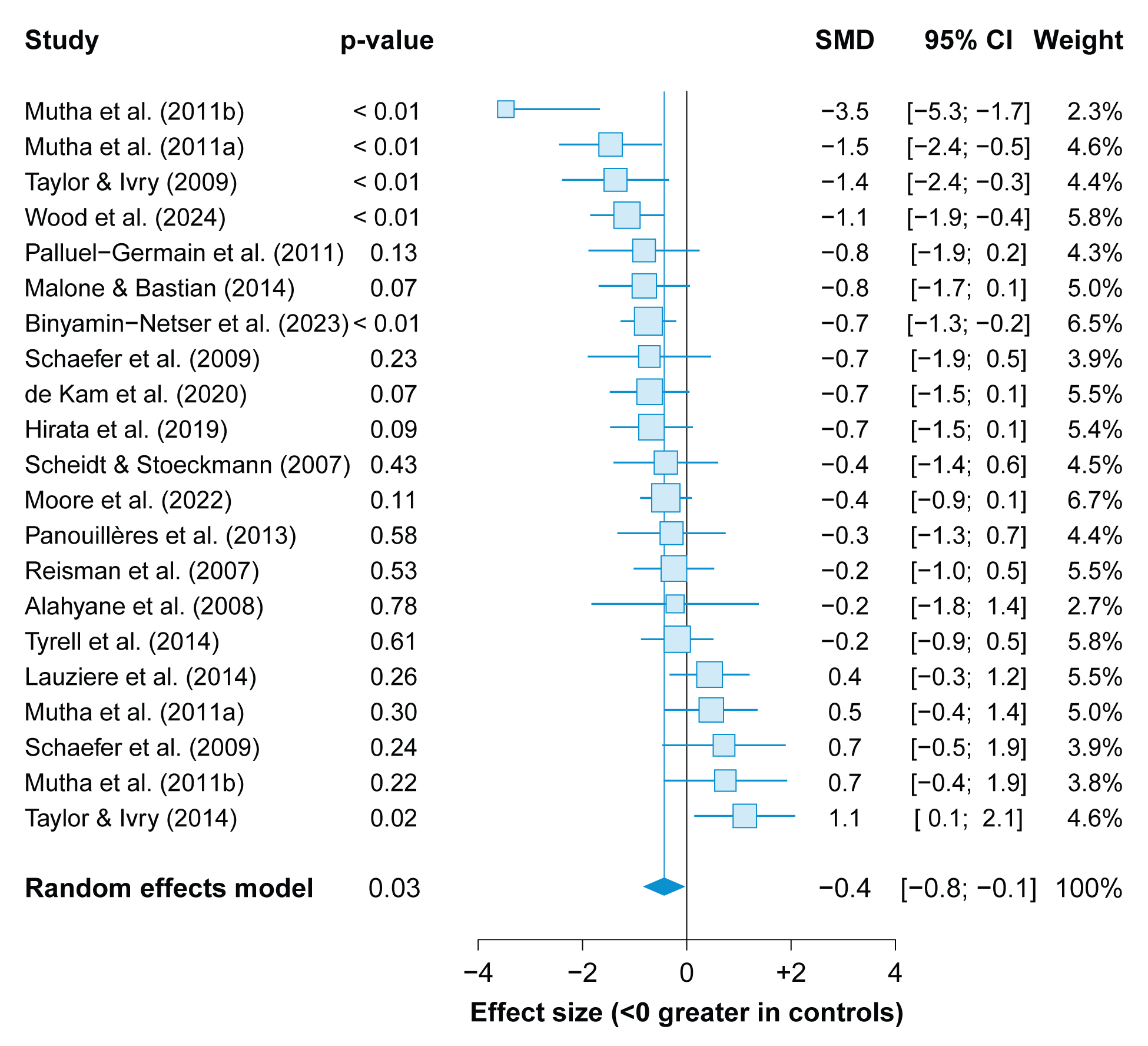


**Figure S2.** Late adaptation. Forest plot comparing the performance of individuals poststroke to neurotypical controls, where negative values indicate greater adaptation in controls (i.e., impaired adaptation poststroke). The overall effect size is indicated by the blue vertical line. Each circle represents a single dataset with its size indicating the weight assigned to that dataset in the random-effects model. Whiskers represent the 95% confidence intervals.

**
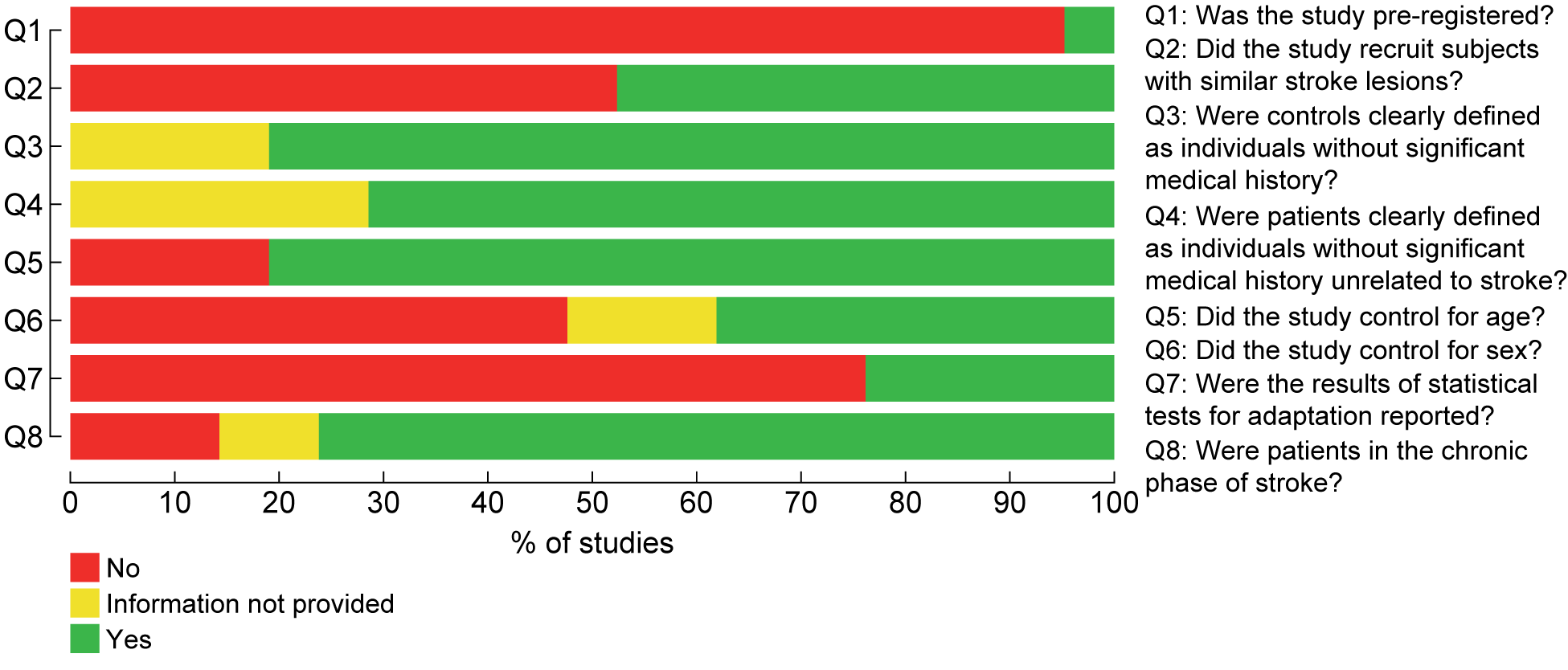
**

**Figure S3.** Risk of bias assessment. Potential sources of bias were evaluated using a modified scale specific to studying motor adaptation in individuals poststroke and neurotypical controls. Green indicates low risk, yellow moderate risk, and red high risk. Each colored section under each question represents the number of studies in that category.

**
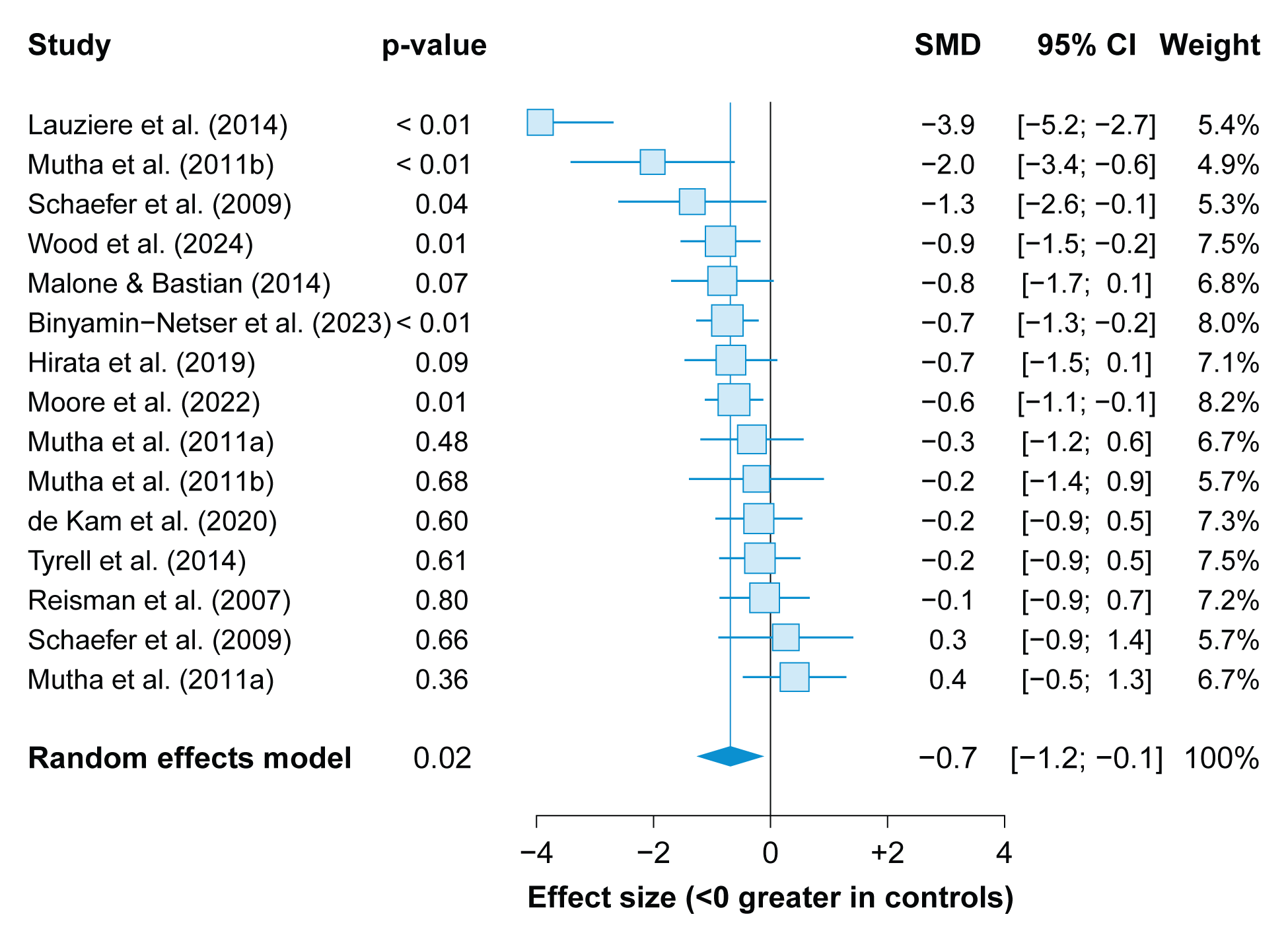
**

**Figure S4.** Forest plot comparing the performance of individuals poststroke to neurotypical controls in the subset of studies with a low risk of bias. We defined studies with a low risk of bias as those that answered 'yes' to at least 4 out of 8 questions in the risk of bias assessment. The overall effect size is indicated by the blue vertical line. Each circle represents a single dataset with its size indicating the weight assigned to that dataset in the random-effects model. Whiskers represent the 95% confidence intervals.

**
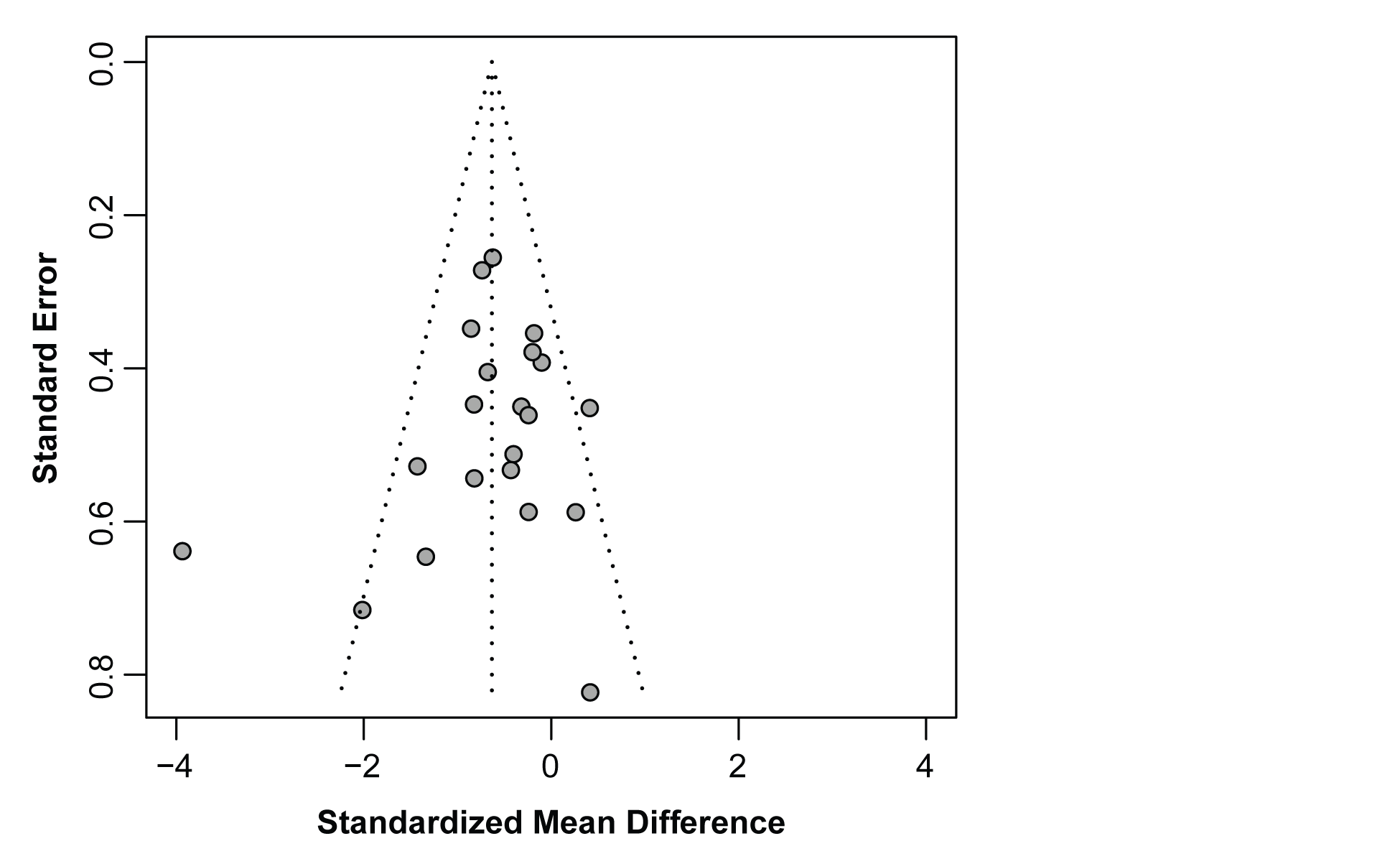
**

**Figure S5.** Funnel plot comparing each study’s effect size on the x-axis and standard error on the y-axis. The dotted lines represent the idealized shape that studies are expected to follow based on the average effect size.

**
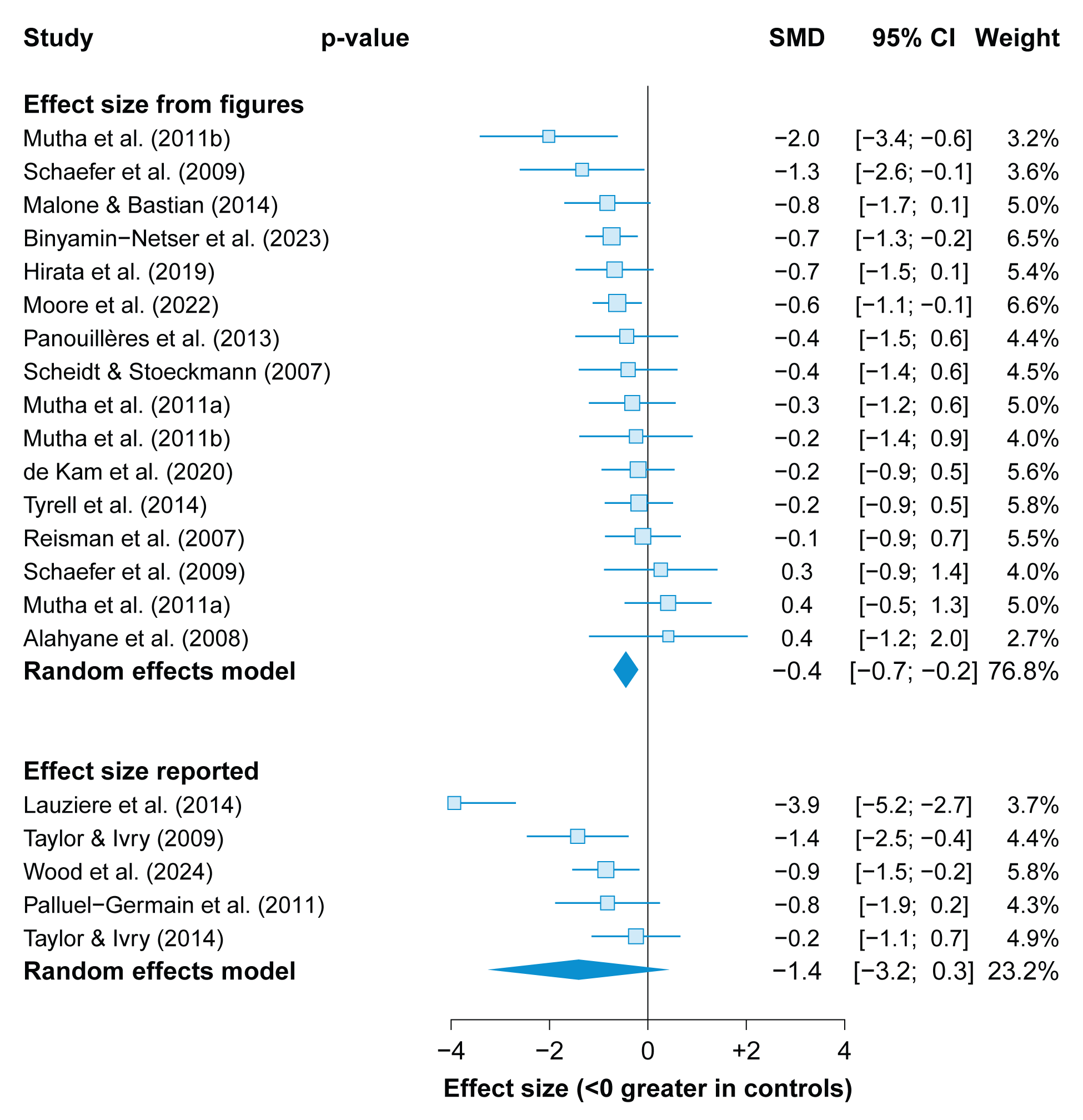
**

**Figure S6.** Impact of data extraction method. Subgroups were assigned based on whether the study reported the effect size directly or whether we calculated it from the figures. Each circle represents a single dataset with its size indicating its weight and whiskers representing 95% confidence intervals.

**
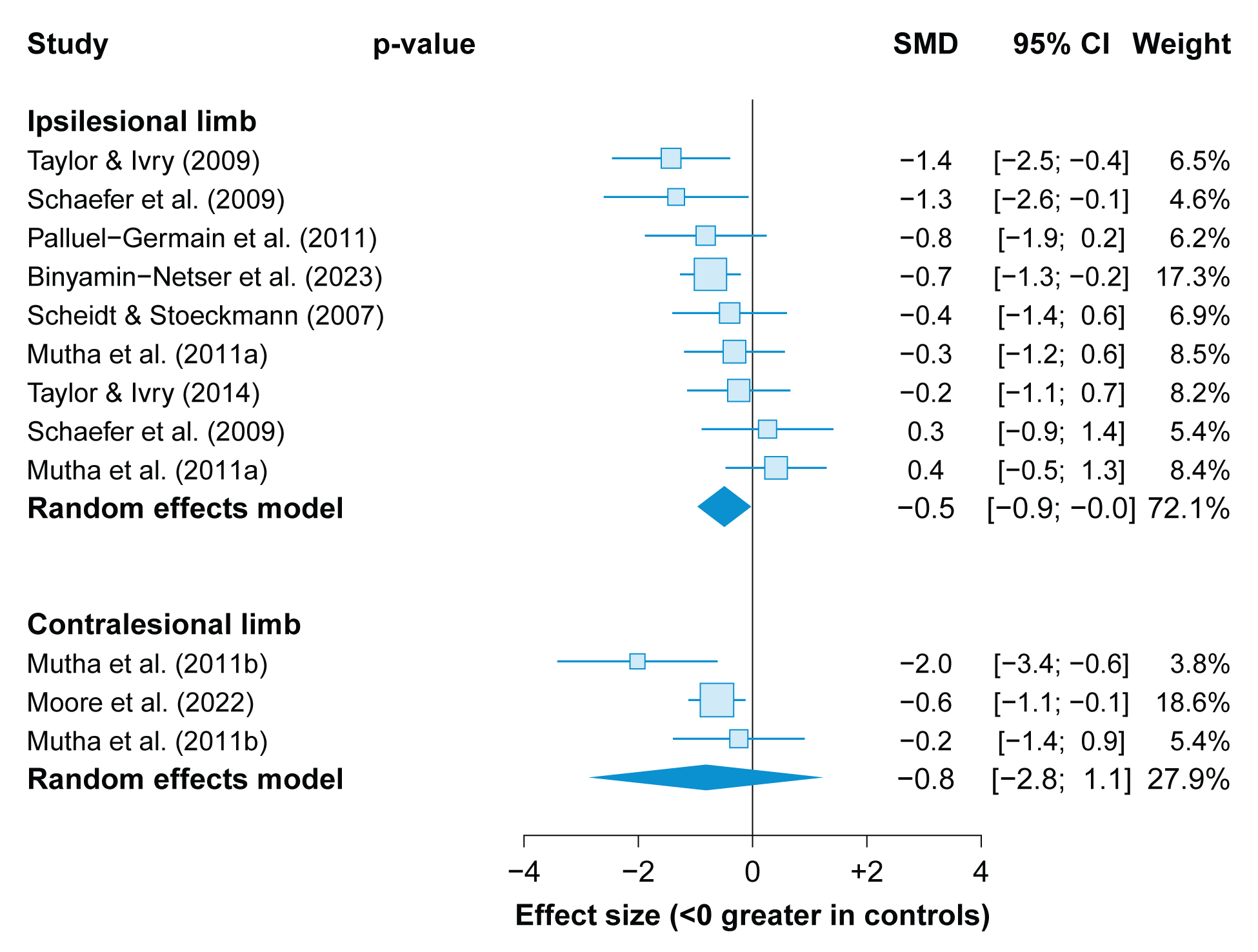
**

**Figure S7.** Impact of patient limb. Subgroups were assigned based on whether patients used their ipsilesional or contralesional limb during the task. Each circle represents a single dataset with its size indicating its weight and whiskers representing 95% confidence intervals.
